# Symbiodiniaceae photophysiology and stress resilience is enhanced by microbial associations

**DOI:** 10.1038/s41598-023-48020-9

**Published:** 2023-11-25

**Authors:** Jennifer L. Matthews, Lilian Hoch, Jean-Baptiste Raina, Marine Pablo, David J. Hughes, Emma F. Camp, Justin R. Seymour, Peter J. Ralph, David J. Suggett, Andrei Herdean

**Affiliations:** 1 Climate Change Cluster, University of Technology Sydney, Ultimo, NSW Australia; 2 Sorbonne University, Paris, France; 3 Australian Institute of Marine Sciences, Townsville, QLD Australia; 4 KAUST Reefscape Restoration Initiative (KRRI) and Red Sea Reseach Centre (RSRC), King Abdullah University of Science & Technology, 23955 Thuwal, Saudi Arabia

**Keywords:** Plant physiology, Abiotic, Symbiosis

## Abstract

Symbiodiniaceae form associations with extra- and intracellular bacterial symbionts, both in culture and in symbiosis with corals. Bacterial associates can regulate Symbiodiniaceae fitness in terms of growth, calcification and photophysiology. However, the influence of these bacteria on interactive stressors, such as temperature and light, which are known to influence Symbiodiniaceae physiology, remains unclear. Here, we examined the photophysiological response of two Symbiodiniaceae species (*Symbiodinium microadriaticum* and *Breviolum minutum*) cultured under acute temperature and light stress with specific bacterial partners from their microbiome (*Labrenzia* (*Roseibium*) *alexandrii*, *Marinobacter adhaerens* or *Muricauda aquimarina*). Overall, bacterial presence positively impacted Symbiodiniaceae core photosynthetic health (photosystem II [PSII] quantum yield) and photoprotective capacity (non-photochemical quenching; NPQ) compared to cultures with all extracellular bacteria removed, although specific benefits were variable across Symbiodiniaceae genera and growth phase. Symbiodiniaceae co-cultured with *M. aquimarina* displayed an inverse NPQ response under high temperatures and light, and those with *L. alexandrii* demonstrated a lowered threshold for induction of NPQ, potentially through the provision of antioxidant compounds such as zeaxanthin (produced by *Muricauda* spp*.*) and dimethylsulfoniopropionate (DMSP; produced by this strain of *L. alexandrii*). Our co-culture approach empirically demonstrates the benefits bacteria can deliver to Symbiodiniaceae photochemical performance, providing evidence that bacterial associates can play important functional roles for Symbiodiniaceae.

## Introduction

Dinoflagellates of the family Symbiodiniaceae are globally distributed from temperate to tropical waters^1^, and establish essential mutualisms with numerous hosts, including cnidarians, poriferans and other protists^2^. When in symbiosis with reef-building corals (Order: Scleractinia), Symbiodiniaceae provide photosynthetically derived carbon^3,4^ and can regulate host tolerance to stress, such as increased sea surface temperatures^5,6^. Consequently, Symbiodiniaceae are essential for the growth and persistence of coral reef ecosystems globally.

The family Symbiodiniaceae is genetically^7^ and functionally diverse^6^, yet what drives this functional diversity remains unclear^8^. Recent studies have conclusively proven that obligate resource exchange between microbial symbionts contributes to the health, physiology, and ecological success of algae, particularly in marine biomes^9,10^. Moreover, bacterial and archaeal associates can contribute to the acclimatisation and adaptation of their microalgal hosts to environmental change through the provision of metabolites or genetic material^11,12^. However, the functional role of bacterial microbiomes in regulating Symbiodiniaceae resource acquisition, competitive performance, and functional diversity, remains unresolved^13–15^. Such unknowns are particularly critical to overcome, given climate change driven amplification of “coral bleaching” events, the process whereby endosymbiotic Symbiodiniaceae are expelled from their hosts^16,17^. Prevailing theory suggests that bleaching occurs via the physiological collapse of the Symbiodiniaceae-cnidarian symbiosis^17–19^, but whether bacteria can facilitate Symbiodiniaceae thermal tolerance is largely unknown.

Symbiodiniaceae can intimately associate with specific bacterial genera, including members of the *Labrenzia*, *Marinobacter* and *Muricauda* genera^13,20^, that can provide metabolites influencing Symbiodiniaceae growth^21^ and stress tolerance^22^. For instance, *Labrenzia alexandrii* and *Marinobacter adhaerens* isolated from Symbiodiniaceae cultures provide the growth stimulant indole-3 acetic acid (IAA) to Symbiodiniaceae^21^. In addition, *Labrenzia* can produce the reactive oxygen species (ROS) scavenging metabolite-DMSP^23^; therefore, the consistent presence of this bacterium in Symbiodiniaceae cultures may support a DMSP-based antioxidant-system in Symbiodiniaceae^24,25^. Notably, *Marinobacter* secrete the siderophore vibrioferrin, which promotes the assimilation of iron in other dinoflagellates, thus supporting optimal microalgal growth and survival in iron-limited conditions^26^. Finally, *Muricauda* has been recently identified as a key intracellular symbiont of 11 species of Symbiodiniaceae^13^, and intriguingly a strain of bacteria (GF1) most closely related to *Muricauda* restored the maximum quantum yield of PSII (as denoted by F_v_/F_m_) and reduced the production of ROS by *Durusdinium trenchii* under heat stress^22^. A similar response has been observed when bacteria-free (axenic) *D. trenchii* was cultured with zeathanxin, a carotenoid with ROS-scavenging abilities produced by the GF1 strain^22^. Thus, it is plausible that *Labrenzia*, *Marinobacter* and *Muricauda*^13^ support Symbiodiniaceae photophysiology and health during stressful conditions (e.g., increased temperature or excess light). While fundamental differences in studying these processes in laboratory cultures versus in situ (such as the physico-chemical landscape of the host^27^) need to be considered, and the relevance of these interactions verified *in hospite,* the presence of these bacteria may be a significant determinant in coral bleaching processes.

Resolving the specific inter-kingdom interactions giving rise to different physiological responses is extremely important given that both Symbiodiniaceae and associated bacteria influence the emergent properties of cultures, including nutrient availability^8,28,29^, metabolite production^21^, reactive oxygen production and quenching^22,30,31^, and thermal tolerance^32^, i.e., physiological benefits that might extend when *in hospite* of corals^14^. Both the biology and analysis of these relationships are inherently complex, but closing these knowledge gaps is possible through the systematic study of Symbiodiniaceae-bacteria interactions^15^ by, for example, first examining how the presence of individual bacteria species affect Symbiodiniaceae fitness when in culture. Here, we report our observations on photophysiological adjustments under temperature and light stress for two Symbiodiniaceae species (*Symbiodinium microadriaticum* and *Breviolum minutum*) in response to specific modifications to the make-up of extracellular bacteria associations (in co-culture with *Muricauda*, *Marinobacter* and *Labrenzia* species) and use this as means to draw new hypotheses underpinning the functional relationship between Symbiodiniaceae and bacteria.

## Results and discussion

To identify the effects of specific bacterial associates on Symbiodiniaceae physiology, we removed all bacteria (extracellular bacteria removed [EBR], using methods and verification processes described previously^13,21^) from two Symbiodiniaceae strains (*Symbiodinium microadriaticum* and *Breviolum minutum*). These two strains were then co-cultured with the bacterial isolates *Labrenzia alexandrii, Marinobacter adhaerens* and *Muricauda aquimarina,* which were originally isolated from these cultures^21^.

**Figure 1:**
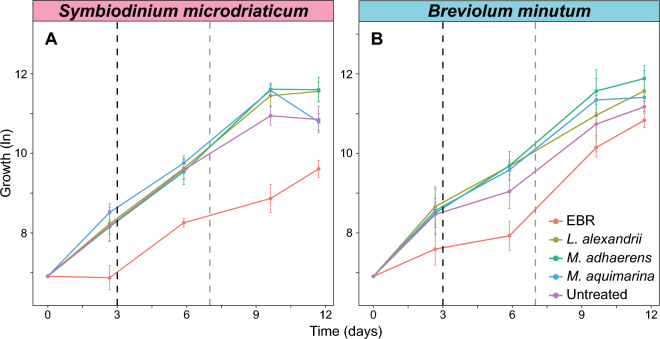
The effect of associated bacteria on the growth of *Symbiodinium microadriaticum* and *Breviolum minutum*. The growth of both (**A**) *S. microadriaticum* and (**B**) *B. minutum* was enhanced by the presence of bacteria compared to extracellular bacteria removed (EBR) cultures. Dotted lines are photophysiological sample time points, pinpointing early- (3 days, black line) and mid-exponential (7 days, grey line) growth phases. Values are natural log of relative chlorophyll intensity *(n* = 4)*.*

We first compared the growth cycle of untreated, EBR (used as extracellular bacteria-free controls) and each of the bacteria co-cultured Symbiodiniaceae (Fig. 1). The specific growth rate (*µ*) of *S. microadriaticum* was reduced by 12.9% when the extracellular bacterial consortium was absent (Fig. 1, Tukey, FDR < 0.01, Table S1), supporting findings that Symbiodiniaceae growth rate in culture is influenced by the presence of specific bacterial consortia^21^. Each bacterial strain remained in culture and substantially increased in abundance during the 12 days (9.2 and 8.3 times more *L. alexandrii,* 22.8 and 1.5 more *M. adhaerens*; and 40.6 and 7.6 more *M. aquimarina* per *S. microadriaticum* and *B. minutum* cells, respectively; Table S2). Each of the three bacterial isolates enhanced the growth of *S. microadriaticum* compared to the extracellular bacteria-free controls (EBR) over the 12-day experimental period (Tukey, FDR < 0.01, Table S1), with growth rates greater than (*L. alexandrii* and *M. adhaerens*) or comparable to (*M. aquimarina*) untreated cultures. While all bacteria isolates induced higher growth rates of *B. minutum* compared to EBR controls, only *L. alexandrii* and *M. adhaerens* significantly enhanced the growth of this species (Tukey, Table S1). This is in line with previous findings revealing data that *L. alexandrii* and *M. adhaerens* support the growth of Symbiodiniaceae, potentially via the provision of the growth promoting hormone indole-3 acetic acid, while no such interaction was detected with *M. aquimarina*^21^. While Symbiodiniaceae heterotrophic feeding on the bacteria^29^ could also have provided a nutritional source leading to an increase in the growth of the algal cells, the concomitant increase in bacterial cell densities suggests these bacteria were able to remain in culture alongside the Symbiodiniaceae (Table S2).

**Figure 2:**
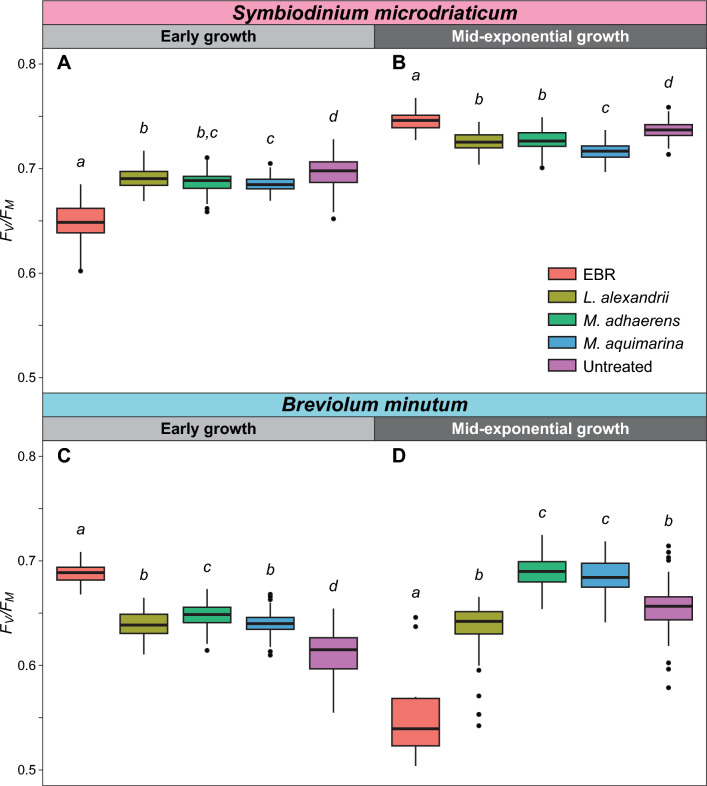
Maximum effective quantum yield (F_v_/F_m_) of modified Symbiodiniaceae-bacteria associations. F_v_/F_m_ measurements were taken from cultures of *S. microadriaticum* (**A** & **B**) and *B. minutum* (**C** & **D**) that had been either untreated*,* had all extracellular bacteria removed (EBR), or co-cultured with either *L. alexandrii*, *M. adhaerens* or *M. aquimarina.* Measurements were taken at early (**A** & **C**) and mid-exponential growth (**B** & **D**) at control temperature (26 °C)*.* Lowercase letters indicate ANOVA post-hoc grouping (*n* = 5).

**Figure 3:**
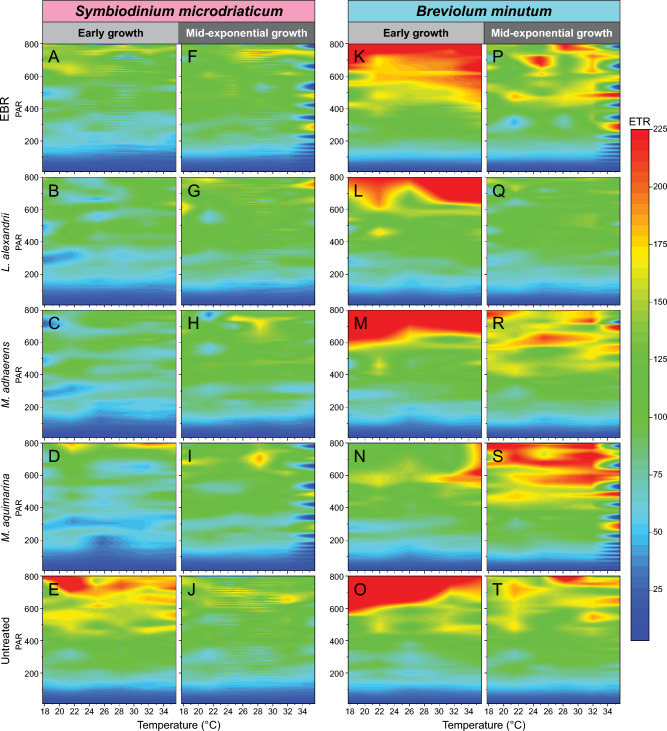
ETR surface plots of Symbiodiniaceae-bacteria co-cultures. *Symbiodinium microadriaticum* (left, **A**–**J**) and *Breviolum minutum* (right, **K**–**T**) were incubated without bacteria (EBR, **A**,**F**,**K**,**L**), or with *L. alexandrii* (**B**, **G**, **M**, **N**), *M. adhaerens* (**C**, **H**, **O**, **P**), or *M. aquimarina* (**D**, **I**, **Q**, **R**), or were left untreated (**E**, **J**, **S**, **T**). The ETR was measure during early- (**A**–**E**, **K**–**S**) and mid-exponential (**F**–**J**, **L**–**T**) growth across temperatures (18–35 °C) and light (0–800 PAR) ranges. Surface plots are scaled to maximum and minimum ETR recorded by the Phenoplate. Grey area indicates readings that exceeded the maximum ETR detection threshold on the Phenoplate. Data available in Table S4.

**Figure 4:**
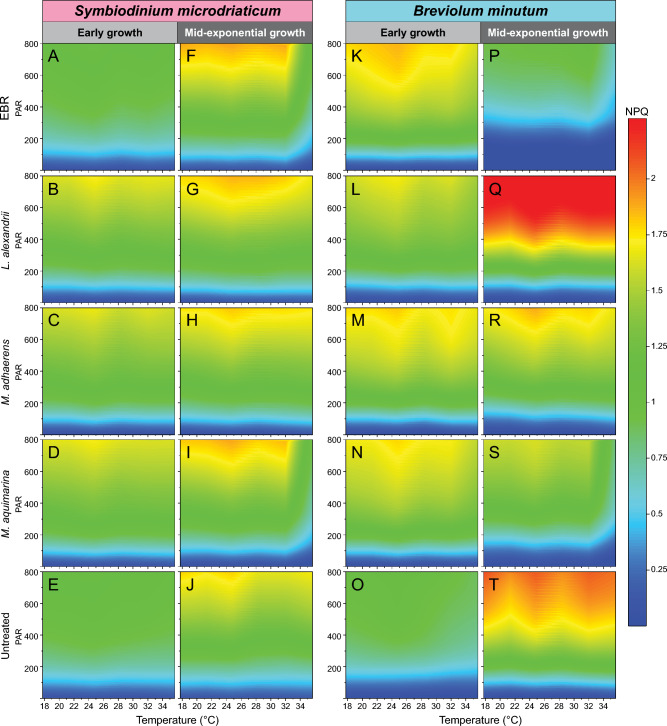
NPQ surface plots of Symbiodiniaceae-bacteria co-cultures. *Symbiodinium microadriaticum* (left, **A**–**J**) and *Breviolum minutum* (right, **K**–**T**) were incubated without bacteria (EBR, **A**,**F**,**K**,**L**), or with *L. alexandrii* (**B**, **G**, **M**, **N**), *M. adhaerens* (**C**, **H**, **O**, **P**), or *M. aquimarina* (**D**, **I**, **Q**, **R**), or were left untreated (**E**, **J**, **S**, **T**). The NPQ was measure during early- (**A**–**E**, **K**–**S**) and mid-exponential (**F**–**J**, **L**–**T**) growth across temperatures (18–35 °C) and light (0–800 PAR) ranges. Surface plots are scaled to maximum and minimum NPQ recorded by the Phenoplate. Grey area indicates readings that exceeded the maximum NPQ detection threshold on the Phenoplate. Data available in Table S4.

**Figure 5:**
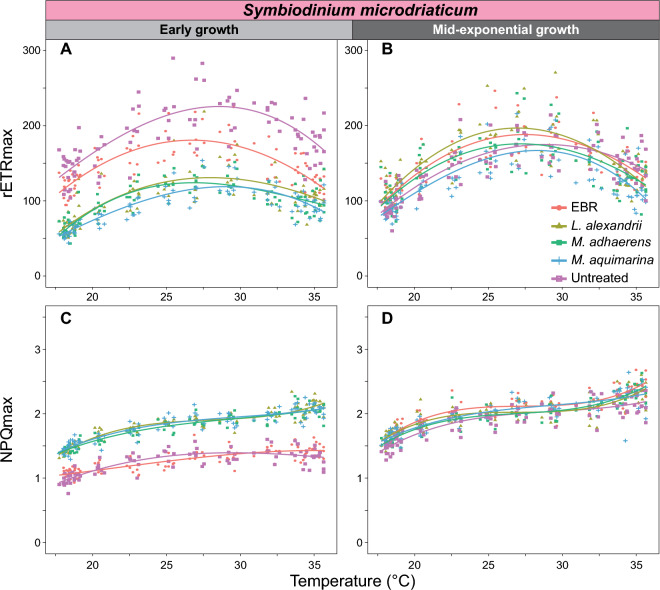
The maximum photochemical and photoprotective capacity of *Symbiodinium microadriaticum*-bacteria associations across temperatures. Maximum relative electron transport rate (rETR_max_; **A** & **B**) and non-photochemical quenching (NPQ_max_; **C** & **D**) at early (**A** & **C**) and mid-exponential growth (**B** & **D**) were calculated across the temperature range (18–35 °C). Polynomial curves were fitted to the plots to represent general trends. *n* = 5 per treatment.

**Figure 6:**
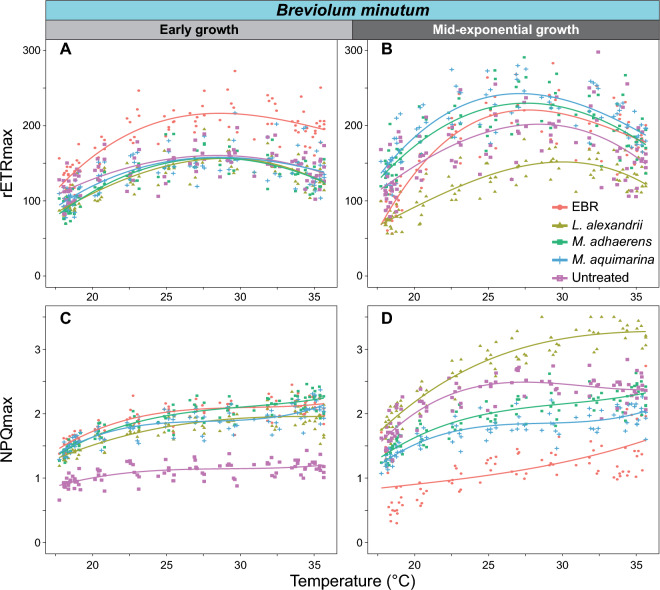
The maximum photochemical and photoprotective capacity of *Breviolum minutum*-bacteria associations across temperatures. Maximum relative electron transport rate (rETR_max_; **A** & **B**) and non-photochemical quenching (NPQ_max_; **C** & **D**) at early (**A** & **C**) and mid-exponential growth (**B** & **D**) were calculated across the temperature range (18–35 °C). Polynomial curves were fitted to the plots to represent general trends. *n* = 5 per treatment.

We examined the photophysiology of the untreated, EBR and bacteria-Symbiodiniaceae co-cultures during early and mid-exponential growth, to capture the effect of bacterial interactions at different Symbiodiniaceae growth stages. We assessed values of maximum photosystem II (PSII) quantum yield (F_v_/F_m_) under control temperature (26 °C) during dark incubation (Fig. 2), and then immediately across a continuous temperature (18.1 – 35.2 °C) and light (7.99–1024.03 μmol photons m^−2^ s^−1^ of actinic broad spectrum white light) gradient in a 96-well plate using the Phenoplate^33^ rapid light curve approach (Figure S1,Table S3), to gather ETR and NPQ per condition combination. We investigated how the different bacteria co-cultures affected the impact of temperature and light combinations on the relative electron transfer rate (rETR = ([Fm' − Ft]/Fm') × PAR × 0.5 × 0.84; Fig. 3, Figure S1). Similarly, we quantified the impact of bacterial co-cultures on the relationship between temperature and dynamic non-photochemical quenching (NPQ = [F_m_ − F_m_´]/F_m_´) (Fig. 4, Figure S1), which represents the extent of heat dissipation by light harvesting pigment complexes associated with PSII to dissipate light energy in excess of photosynthetic capacity. Both the maximum relative electron transport (rETR_max_) and maximum non-photochemical quenching capacity (NPQ_max_) were determined across the temperature range for each co-culture, and fitted to a polynomial distribution (Figs. 5, 6).

The maximum PSII quantum yield (F_v_/F_m_) of *S. microadriaticum* was higher in the presence of *Muricauda aquimarina*, *Marinobacter adhaerens*, and *Labrenzia alexandrii* during early exponential growth at control temperature when compared to EBR cultures (Tukey’s test, FDR < 0.05; Fig. 4A). This corresponds with recent evidence from microbiome-manipulated cultures of a Symbiodiniaceae isolate from the genus *Cladocopium*^34^, which displayed an opposite response, where F_v_/F_m_ was reduced and *Muricauda* and *Marinobacter* sp. disappeared from the microbiome after three days (the same time point as the early growth stage sampled here) under both control and light stress conditions. Similarly, F_v_/F_m_ of *Breviolum minutum* cultures in mid-exponential growth were increased when in co-culture with *M. aquimarina, M. adhaerens* and *L. alexandrii* (Tukey’s test, FDR < 0.05; Fig. 2D). Furthermore, *B. minutum* cultures retained a consistent rETR even at high temperatures and high PAR, when co-cultured with these bacteria (Fig. 3L,M,N,O,P,Q,R,S,T). Values of F_v_/F_m_ continued to change over time for all the bacteria co-cultures of both Symbiodiniaceae species (Fig. 2), although only *S. microadriaticum-M. aquimarina* and *B. minutum-M. adhaerens* yielded significant increases in F_v_/F_m_ between days 3 and 7 of growth (ANOVA, FDR < 0.001; Table S5 and S7).

The close relationship between Symbiodiniaceae and bacteria from *Muricauda, Labrenzia* and *Marinobacter* genera is becoming increasingly evident, with multiple theories on the nature of their mutualistic interactions^22,32,35^. One possible class of compounds underpinning these interactions is the bacterial production of siderophores, which can chelate iron and provide Fe(III) to their microalgal partners^36,37^. Several species of *Marinobacter* sp. produce the low-affinity siderophore vibrioferrin (VF) to promote the growth and survival of their dinoflagellate partner in nutrient-limited environments^26,38^, and some *Labrenzia* species also produce siderophores^38^. Photobiological health is widely dependent on iron availability, where iron is used in PSII reaction centres (in the electron chain), thylakoid membrane repair and maintenance and reactive oxygen species (ROS) dissipation^39–41^. Symbiodiniaceae often inhabit oligotrophic waters, with trace levels of iron^41^, yet generally require large amounts of iron to reach maximum growth in culture relative to other dinoflagellates^37^. Indeed, iron availability has recently been shown to govern growth performance under heat stress in isolates of *Breviolum*^42^. Thus, increase in iron bioavailability through the presence of bacterial siderophores could explain the higher maximum photosynthetic capacity observed in the bacteria co-cultures. To examine the siderophore production by our bacteria isolates, we used a Chrome Azurol S (CAS)-based assay^43^. CAS is a metal-sensitive, colourimetric dye that changes from blue when iron is present, to yellow when iron is absent. Positive siderophore production is observed by yellow to orange colouration surrounding plated bacteria colonies. Both *L. alexandrii* and *M. adhaerens* presented yellow halos, indicating these strains produce siderophores, while no yellow colouration was observed for *M. aquimarina* (Figure S1).

The strong negative photophysiological response in both light- (rETR) and dark-acclimated (F_v_/F_m_) conditions observed for the EBR cultures versus *B. minutum*-bacteria co-cultures and untreated cultures during mid-exponential growth (Figs. 2C,D; 3 K-T) may be indicative of reduced ability to store intracellular iron in *Breviolum minutum* compared to other Symbiodiniaceae species, including other *Breviolum* species^37,42^. As iron availability decreases in the medium over time^40^, there may have been a greater reliance on the bacteria presence for iron acquisition by mid-exponential growth (and could explain the demise of the *B. minutum* EBR cultures), thereby strengthening the link between iron acquisition through bacterial siderophores in reef ecosystems and photobiological health. Meanwhile the absence of a similar reduction in F_v_/F_m_ in EBR cultures of *S. microadriaticum* by mid-exponential growth may be due to this species’ ability to use other trace metals in substitution of iron, for example alter copper efficiency to assist thylakoid maintenance via iron–copper replacement during iron limitation^37,44,45^, or metal-independent processes (e.g. Fe-dependent electron transport ferredoxin to Fe-independent flavodoxin^46^).

Whilst bacterial presence appeared to elicit a beneficial response on F_v_/F_m_ for *S. microadriaticum* cultures during early growth, it also ultimately reduced rETR across light and temperature stress compared to EBR cultures. Based on the heat maps (Fig. 1A,B,C,D), values of rETR (and hence effective operating efficiency) for EBR control cultures were found to be consistently slightly elevated compared to the co-cultures at higher PAR (~ 650–800 μmol photons m^−2^ s^−1^) and EBR culture rETR_max_ was consistently higher across the temperature range compared to the bacteria co-cultures (Table S6A, Anderson–Darling (A-D) test, *p* < 0.001; Fig. 5C). This suggests that under dark-acclimated conditions, the presence of these three bacteria support higher photosynthetic efficiency at control temperature, but under steady state photosynthetic lighting conditions, the absence of extracellular and co-cultured bacteria in *S. microadriaticum* cultures yields higher photosynthetic efficiency across temperatures at this growth stage.

Similarly, EBR cultures had higher values of rETR_max_ across the temperature range compared to the bacteria co-cultures and untreated cultures during early growth in *B. minutum* (Fig. 5C). It is not clear why the presence of bacteria in *S. microadriaticum* cultures reduced the observed rETR relative to the bacteria free cultures. One possibility is that, for *S. microadriaticum*-bacteria co-cultures, competition for resources that regulate photosynthetic rates reduced the rETR in these Symbiodiniaceae cultures, and EBR cultures might require higher photosynthetic efficiency to support growth^47,48^. The rETR/ rETR_max_ values continue to be higher in *S. microadriaticum* EBR than all bacteria co-cultures (except for *S. microadriaticum*-*L. alexandrii* rETR_max_ between 18 ~ 32 °C) across the different growth phases, supporting the hypothesis that EBR cultures increased actual photosynthesis to maintain growth across the cycle, although clearly at significantly reduced growth rates (Fig. 1A), but resolving this will require further targeted examination.

Of the three bacteria isolates tested here, association with *M. aquimarina* exerted the strongest positive effect on *B. minutum* in terms of increased F_v_/F_m_, rETR (particularly at high PAR, and hence and rETR_max_) across temperatures versus all other cultures at mid-exponential growth (Figs. 2D and 3R,S). *M. aquimarina* therefore appears to be an advantageous bacterial associate of *B. minutum* in terms of supporting both growth and photochemical capacity when in culture. Whether this interaction persists *in hospite* and across Symbiodiniaceae genera remains to be evaluated. Importantly, *Muricauda* was recently identified as a key intracellular symbiont in 11 different species of Symbiodiniaceae, including *B. minutum, S. microadriaticum,* and genera of Symbiodiniaceae that form associations with corals^13^. Interestingly, however, at temperatures above 32 °C, rETR appears to reduce for both Symbiodiniaceae species associated with *M. aquimarina* across a range of PAR (Fig. 3S). Similarly for *S. microadriaticum*, while the ETR and rETR_max_ distributions at both time points were similar across the bacteria tested (Fig. 3A,B,C,D,E,F,G,H,I,J, and 5A,B), *L. alexandrii* co-cultures exhibited slightly higher rETR_max_ values from 18 ~ 32 °C, however, this benefit was reduced at temperatures > 32 °C, where the untreated cultures had the highest rETR_max_ response (Fig. 4D).

Interactions between bacteria and microalgae can be influenced by environmental conditions^49^. As such, it is plausible that the functional role of *Muricauda* or *Labrenzia* sp. associates change under thermal stress, where the Symbiodiniaceae switch cellular investment from normal photophysiology and growth processes, to survival^6^. Indeed, a recent study found that a close relative of *Muricauda* translocated the antioxidant Zeaxanthin^18^. In addition, *Labrenzia* sp. can also play a role in ROS scavenging^23^, and Symbiodiniaceae taxa are widely reported as producers of various ROS under heat stress^8,50,51^. Thus, bacterial functions may shift at 32 °C from one of siderophore action (and thus photophysiological support), to one of antioxidant provision. Indeed, elevating the iron content in cultured medium has been found to lead to higher photosynthetic performance of Symbiodiniaceae when exposed to temperature stress^42^, perhaps as bacteria functional roles redirect to antioxidant provision. Nevertheless, requirements of iron in Symbiodiniaceae appear higher under thermal stress^52^ and iron deficiency is also documented to increase ROS scavenging in the Symbiodiniaceae genus *Fugacium*^53^, so whether any changes in bacteria functional roles are transient or optimal for the Symbiodiniaceae remains to be fully resolved.

Growing evidence points towards the significant contribution of the coral holobiont towards stress mitigation and aided survival of Symbiodiniaceae^54,55^. NPQ is a mechanism employed by photosynthetic organisms to dissipate excess absorbed light energy as heat, to ultimately prevent damage to the photosynthetic machinery to retain photochemical activity^16,56^. During early growth of *S. microadriaticum*, both EBR and untreated cultures had similar NPQ patterns across light intensity and temperature (Fig. 3A,E), but bacteria co-cultures exhibited higher NPQ when PAR was > 200 μmol photons m^−2^ s^−1^ and with comparable distributions across the range tested (Fig. 3B,C,D). At both time points, NPQ_max_ shows a pattern in which changes in temperature beyond the normal thermal range trigger NPQ mechanisms, as is common amongst Symbiodiniaceae undergoing temperature and/or light stress^57,58^. During early growth of *S. microadriaticum*, the distribution of NPQ_max_ across temperatures was statistically similar for untreated and EBR cultures, both of which were lower than all three bacteria co-cultures (Table S6B, A-D test *p* < 0.05; Fig. 4A,E). However, at mid-exponential growth, NPQ was elevated across the temperature range in EBR and untreated cultures, and – as with rETR_max_ distributions – became more similar to those of bacterial co-cultures, which had further stabilized across temperature and light (Fig. 3F,G,H,I,J). Similarly, NPQ_max_ increased for all *S. microadriaticum* cultures, with no significant differences across temperature treatments, except between untreated and EBR cultures (Fig. 6E,F; Table S6A,B,C,D test *p* < 0.05).

Similar to *S. microadriaticum* co-cultures, *B. minutum* co-cultures had elevated NPQ values at PAR > 400 μmol photons m^−2^ s^−1^, and with comparable distributions across the range tested, compared to untreated *B. minutum* cultures during early growth (Fig. 4M,N,O,P,Q,R,S). Also, the three *B. minutum* bacteria co-cultures exhibited higher NPQ_max_ distributions across temperatures compared to the untreated cultures during early growth, although EBR was more closely matched to the bacteria co-cultures than untreated cultures (Fig. 5E). During early growth, EBR cultures had the highest NPQ values at PAR > 400 μmol photons m^−2^ s^−1^, but by mid-exponential growth, NPQ across temperature and light (and NPQ_max_ distributions across temperatures) were lowest for EBR cultures, supporting a marked decline in culture health, as also reflected by F_v_/F_m_ and rETR_max_ (Fig. 3L). The highest NPQ values above 200 μmol photons m^−2^ s^−1^, and NPQ_max_ distributions across temperatures were detected for *L. alexandrii* co-cultures, particularly at higher temperatures (> 30 °C) (Fig. 5F), indicating strong photoprotection by *L. alexandrii* for *B. minutum* cultures. *M. aquimarina* and *M. adhaerens* co-culture values of NPQ_max_ distributions across temperatures were below those for untreated cultures, but above EBR, and NPQ_max_ values for *M. adhaerens* and untreated cultures coincided at the highest temperatures (> 35 °C) (Fig. 4F). Between time points, *M. adhaerens* NPQ_max_ distributions remained relatively constant, but significantly changed for all other cultures (A-D test, *p* < 0.001; Table S7).

Breakdown of photobiological activity typically leads to ROS accumulation within Symbiodiniaceae cells^22,50,59^ and their hosts^17,60^, emphasising the importance of protective mechanisms such as NPQ and the xanthophyll cycle^61^. The xanthophyll pigment zeaxanthin has been identified as a potential thermal tolerance aid to dinoflagellates, including Symbiodiniaceae^22^, and has been isolated from bacterial strains closely related to *Muricauda*^22^. Zeaxanthin is a functional equivalent of diadinoxanthin and diatoxanthin, two pigments that scavenge ROS and protect photosynthetic organisms via NPQ generation from lipid peroxidation and oxidative stress^62–67^. Whilst the exact dynamics between the NPQ and xanthophyll photoprotective mechanisms are poorly understood, evidence suggests NPQ responds proportionately to the presence of diatoxanthin in other species of algae^22,62,64^. Given the functional similarities between the two xanthophyll pigments, it has been postulated that dinoflagellates can convert zeaxanthin to diatoxanthin, which would induce NPQ under stress conditions, as provided by their bacterial symbionts^68,69^. Bacteria in the genus *Muricauda* are characterised by the production of zeaxanthin, which therefore potentially explains the NPQ response of *B. minutum-M. aquimarina* cultures relative to the EBR cultures.

Values of NPQ_max_ for *B. minutum* in the presence of *M. aquimarina* were lower than the co-culture with the other two bacteria. Different bacteria can contribute different antioxidants (with varying antioxidant capacities) to affect microalgal photophysiology^22,23,28^, and this may explain the patterns observed in *B. minutum*. *L. alexandrii* induced an extremely elevated NPQ response across the temperature gradient during mid-exponential growth in *B. minutum* cultures. A notable function of *Labrenzia* is its ability to produce ROS scavenging-DMSP. We confirmed the production of DMSP by our *L. alexandrii* isolates via LC–MS analysis (Table S8). Therefore, the consistent presence of this bacterium in Symbiodiniaceae cultures points to the role of a DMSP-based antioxidant-system within its interaction with Symbiodiniaceae. Evidence suggests that the high presence of DMSP within coral ecosystems is linked to antioxidant action within Symbiodiniaceae, scavenging high levels of ROS which are produced under thermal and light stress^23,70–72^. Recent studies have identified the ability of prokaryotes to contribute DMSP within cnidarian hosts when undergoing temperature and light stress^72^. In addition, *Labrenzia sp.* has been strongly associated with production of DMSP and the relative abundance of this bacterium in the microbiome of *Breviolum* can shift under thermal stress^15,32^. *B. minutum* NPQ responses in co-culture with *L. alexandrii* suggest a link between increased thermal and light stress, and strong dissipation at perhaps an earlier temperature- and light-stress threshold compared to other co-cultures (e.g., > 30 °C and > 400 μmol photons m^−2^ s^−1^). The DMSP produced by *Labrenzia* sp*.* may be a fast-acting molecule to induce algal NPQ^74^, as the cultures were only exposed to short bursts of thermal stress allowing little time for microbial community adjustment.

In summary, Symbiodiniaceae demonstrate enhanced growth and retained photochemical performance under acute heat stress when associated with these bacteria. This corroborates the hypothesis that persistence of Symbiodiniaceae in their free-living state is supported by bacterial presence^15^, although whether these associations exist beyond laboratory cultures to the open ocean and on reefs, remains to be resolved. Symbiodiniaceae can make stable associations with extra- and intracellular and bacterial symbionts, whether cultured or *in hospite*, including the genera tested here^13,20^, and it is becoming increasingly clear that bacterial microbiomes play an important role in regulating Symbiodiniaceae growth competitive performance and functional diversity^8,21,22,32,75,76^. If these associations and functional roles persist *in hospite* and in other Symbiodiniaceae genera, they may in turn be central to the health of corals. Incorporating these interactions when predicting the resilience and adaptability of coral reefs to environmental change is critically important, and potentially supports new conservation and restoration approaches, e.g., via probiotics^15^. For example, the production of siderophores by *Marinobacter* sp. to help bind and concentrate iron into bioavailable forms might support Symbiodiniaceae photophysiology when free-living^26^ and as coral endosymbionts. Similarly, a link has been suggested between the relative abundance of *Labrenzia* sp., and Symbiodiniaceae composition and thermal tolerance of corals^15,54^; thus if *Labrenzia* sp. aid the ROS-scavenging and hence thermal tolerance of Symbiodiniaceae, this may extend to *in-hospite* Symbiodiniaceae, and could aid against temperature-induced bleaching. Our findings therefore highlight the importance of examining and incorporating multipartner interactions when evaluating coral phenotypic responses to environmental change^15,77^, but unpacking the nature of these interactions will be critical in understanding how bacterial partnerships support Symbiodiniaceae survival both in their free living stages and in symbiosis.

## Methods

### Symbiodiniaceae cultures

Two Symbiodiniaceae species were used from existing stock collections at the University of Technology Sydney, *Symbiodinium microadriaticum* (ITS2: A1, culture ID: RT61, from *Cassiopeia xamachana*), and *Breviolum minutum* (ITS2: B1, culture ID: RT2, CCMP2463, from *Exaiptasia pallida*)^20,32^. Each Symbiodiniaceae species was sub-cultured (*N* = 5 per Symbiodiniaceae species) by adding 10 mL of original cultures in 90 mL of autoclaved and filter sterilised (0.22 µm) artificial seawater (ASW) supplemented with F/2 nutriments. The medium was not replaced for the duration of the growth cycle. Subcultures were grown for one month to achieve a cell density of 10^6^ cells/mL at 26 °C with an irradiance of 85 ± 15 µmol photons m^−2^ s^−1^ (Philips TLD 18W/54 fluorescent tubes, 10,000 K on a 12 h:12 h light:dark cycle).

### Antibiotic treatments

External bacteria removal and antibiotic treatments were conducted as per Matthews et al. (19). To each EBR or co-culture treatment subculture (*N* = 16 per Symbiodiniaceae species), TritonX-100 was added to a final concentration of 20 µg/mL and placed on a shaker at mid speed for 30 s. All subcultures (treatment and control) were immediately centrifuged at 700 × *g* for 10 min at 26 °C, the supernatant discarded. This was repeated twice more, after which untreated cells were rinsed in 20 mL ASW only and centrifuged at 700 × *g* for 10 min at 26 °C. Cells were resuspended in 9 mL ASW + F/2 and transferred to sterile culture flasks.

To each EBR or co-culture treatment subculture (*N* = 16), cells were resuspended in 20 mL ASW, filtered on a 0.22 µm Durapore® membrane filter, and rinsed with 20 mL 4% sodium hypochlorite. Cells were transferred to sterile culture flasks and 1 mL of antibiotic Mix 1 (Table S9) was added, and 1 mL ASW added to each control (*N* = 4), and 9 mL ASW + F/2 added to all cultures and flasks were replaced in the incubator. After 48 h, an additional 30 mL ASW + F/2 was added to all cultures. Cultures were allowed to recover for five days, and this process was repeated four times. After 1 week, bacteria absence was confirmed by two individual tests: plating 1 mL of each Symbiodiniaceae culture on Marine Agar followed by 5 days incubation and visual checks for bacteria contamination, and SYBR Green staining and absence of signal during flow cytometry following blank correction (Table S10; Fig S3c,d; see *Symbiodiniaceae and bacterial cell density analysis* below).

### Bacterial isolates

Pure cultures of *Labrenzia alexandrii, Marinobacter adhaerens, and Muricauda aquimarina* bacteria were obtained from pure glycerol stock cultures as per Matthews et al. (19). To reanimate the bacterial cultures, a sterile loop was used to pick from the glycerol stock and spread onto 100% marine agar for 5 days. Approximately 2–3 colonies were picked and inoculated in 50 mL 50% Marine Broth + ASW for 12 h until bacteria cells were in late exponential growth (as confirmed via pilot studies).

### Symbiodiniaceae and bacterial cell density analysis

Flow-cytometric cell abundance was conducted for each Symbiodiniaceae and bacteria isolate culture immediately before co-culture mixing. For each Symbiodiniaceae culture (*N* = 20 per species), a 100 µL aliquot was collected, diluted to 1:10 with ASW and fixed in glutaraldehyde (Sigma-Aldrich; 2% final concentration) for 15 min. Samples were directly used for flow cytometry analysis (CytoFLEX S, Beckman Coulter, CA, United States) to assess concentration of Symbiodiniaceae cells and relative cell chlorophyll fluorescence (650 nm) and side scatter (SSC). Sample blanks (*N* = 5) were run alongside and the average bacteria count from the blanks subtracted from each sample count (blank correction). Symbiodiniaceae flow cytometer gating strategy is shown in Figure S2a.

Total prokaryotic abundances were quantified by staining the cells with SYBR Green (1:10,000 final dilution) and analysis on a CytoFLEX S (Beckman Coulter, CA, United States) flow cytometer with filtered MilliQ water as the sheath fluid. For each sample of each culture, 3 × 200 µL aliquots were taken at the time of sampling and fixed in glutaraldehyde (Sigma-Aldrich; 2% final concentration). The samples were analysed at a flow rate of 10 μL min^–1^, with bacterial cells discriminated according to forward scatter (FSC), side scatter (SSC), and green fluorescence (SYBR Green, 488 nm). Sample blanks (media only; *N* = 5) were stained and run alongside, and the average bacteria count from the blanks subtracted from each sample count (blank correction; Figure S2c). Bacteria flow cytometer gating strategy is shown in Figure S2b.

### Symbiodiniaceae-bacteria specific co-culture generation

Before use, all Symbiodiniaceae subcultures were rinsed by centrifugation at 700 × *g* for 10 min at 26 °C, the medium discarded, and resuspended to a concentration of 10^5^ cells / mL in fresh ASW + F/2 medium. From each subculture, 50 mL was transferred to sterile 250 mL glass conical flasks. To create bacteria co-culture treatments (per Symbiodiniaceae species, *N* = 4 each of *Labrenzia* sp., *Marinobacter* sp., and *Muricauda* sp.), 10^4^ cells of the respective bacteria were added to achieve a final ratio of algae:bacteria of 10:1. In addition, *N* = 4 subcultures of each of bacteria-free (antibiotic treated but without bacteria addition) and untreated (no antibiotic treatment) per Symbiodiniaceae species were maintained. Bacteria in the co-cultures were quantified via flow cytometry (as described above) at the end of the growth period and normalised to Symbiodiniaceae cell density.

### Symbiodiniaceae growth

For each Symbiodiniaceae species and co-culture mix (including bacteria-free and untreated subcultures), 2 × 400 µL aliquots were placed into a 48-well plate (*N* = 2 wells per co-culture). Plates were sealed with electrical tape and placed in an incubator at 26 °C with an irradiance of 70 ± 5 µmol photons m^−2^ s^−1^. Growth rates were estimated by measuring in vivo chlorophyll *a* fluorescence (relative fluorescence units) in a Tecan Spark plate reader^21^ and measured as follows; 16 reads per well at excitation wavelength of 455 nm, emission wavelength of 630, 664, and 750 nm, gain at 80 nm; with 30 flashes at a frequency of 400 Hz; integration time of 20 µs; lag time of 0 µs, and settle time of 10 ms. Measurements were taken at the start (0), 2.7, 5.9, 9.6 and 11.7 days after incubation, and plates replaced in incubators immediately after each measurement (~ 30 s per plate). Specific growth rates (*μ*) were calculated from the linear regression of the natural log of the in vivo fluorescence versus time. Standard deviation of *μ* was calculated from *μ* values from biological replicates (*N* = 4) over the growth period. Percentage growth enhancement was calculated as the difference between *μ*_co-culture_ and *μ*_EBR_ divided by *μ*_co-culture_. Specific growth rates were compared between treatments using ANOVA and Tukey’s post-hoc.

### Phenoplate phenotyping analysis

The phenoplate Rapid Ligh Curve (RLC) method was carried out according to Herdean et al. 2023^71^, modified as follows (Fluorescence tracings for this experiment shown in Figure S2). The protocol began with a one-minute incubation period, in which far-red preillumination was applied (730 nm) and temperature of cultures was maintained at 26 °C. This was followed by a saturating pulse for determination of F_v_/F_m_ (Figure S2, Fig. 2). This was followed by five minutes of low light at 10.9 μmol photons m^−2^ s^−1^ and temperature ramping (Steady state temperatures after ramping shown in Table S3). The final step was the RLC^78^, performed using 15 increasing illumination steps of broad spectrum white actinic light running for 30 s each from 35.05 to 1024.03 μmol photons m^−2^ s^−1^ (Light intensities of each step shown in Table S4). The Anderson–Darling test was performed to identify any significant differences in the shape of the distributions across temperatures between co-cultures and over time. To assess whether the optical properties of the sample—altered due to the presence of bacteria—influenced the PAM measurements, we evaluated F_v_/F_m_ ratios across different mixtures of Symbiodiniaceae and bacteria immediately post-mixing. Our observations indicated no significant difference in the PAM measurements between samples containing bacteria and those with sterile media only (Table S11).

### DMSP extraction from L. alexandrii

Pure cultures (*N* = 3) of *L. alexandrii* was reanimated as described above in 50 mL 50% Marine Broth (Difco) + ASW + 5 mM methionine for 12 h. Samples were centrifuged at 1,500 × g for 10 min, and the medium removed. Cells were snap frozen in liquid nitrogen, and resuspended in 4 mL 80% methanol and sonicated on high for 20 min at 4 °C. Cells were pelleted at 5,000 × g for 10 min, and the methanol extract supernatant collected. Each methanol extract was dried under a nitrogen stream, using a sample concentrator (Ratek dry block heater). Samples were then resuspended into a fixed methanol volume (1 mL). This normalisation step was needed to (i) account for slight differences in extract volumes between samples, and (ii) concentrate each sample before chemical analyses. The resuspended samples were centrifuged to remove particulates (20 min at 15,000 rpm), the supernatants were transferred to 1 mL glass tubes, and kept at − 20 °C until LC–MS analysis.

### Quantification of DMSP by LC–MS

LC–MS was used to confirm the production of DMSP by *L. alexandrii*. LC–MS was carried out using an Ultra High Performance Liquid Chromatography (UHPLC; 1290 Infinity II LC System and a Waters Acquity UPLC BEH Hilic column (2.1 × 100 mm with a particle size of 1.7 μm)). Mass spectrometry spray chamber conditions were as follows: gas flow = 15.0 l/min, gas temperature = 225 °C, chamber current 0.21 μA and capillary current = 46 nA. Solvent A was 0.1% ammonium formate in water and solvent B was 0.1% ammonium formate in acetonitrile. A seven-point calibration curve was performed for quantification of DMSP, using DMSP solubilised in the extraction solvent (i.e., methanol).

### CAS assay for siderophore production

CAS agar medium was prepared according to the step-by-step procedure described in Louden et al. 2011^43^. Pure cultures (N = 3) *of L. alexandrii, M. adhaerens* and *M. aquimarina* were inoculated in 5 mL MM9 medium and incubated for 14 h. After that time, 10 µL of each bacteria culture spread onto individual CAS plates. Plates were grown at 26 °C until bacteria colonies were visible. In separate CAS plates, 10 mL of 50 µM EDTA was spread as a positive control and 10 mL of sterile MM9 as a negative control. The isolates showing yellow to orange coloured ring around the colonies were then considered as positive siderophore producing strains (Figure S1).

### Supplementary Information

Supplementary Information 1.

Supplementary Information 2.

## Data Availability

All data generated or analysed during this study are included in this published article and its Supplementary Information excel file.
