# Supplementary material for: Symbiodiniaceae photophysiology and stress resilience is enhanced by microbial associations
Source: Sci Rep. 2023 Nov 25;13:20724. doi: 10.1038/s41598-023-48020-9 (PMC10676399; doi:10.1038/s41598-023-48020-9)
Supplement: Supplementary file 2 — Supplementary Information 2. [file 41598_2023_48020_MOESM2_ESM.docx]

**Supplemental Information**

**for**

**Symbiodiniaceae photophysiology and stress resilience is enhanced by microbial associations.**

Authors: Jennifer L. Matthews, Lilian Hoch, Jean-Baptiste Raina, Emma F. Camp, Justin R. Seymour, David J. Hughes, Peter J. Ralph, David J. Suggett, Andrei Herdean

**Figure S1. Iron chelating CAS assay plates for the detection of siderophore production by cultured *L. alexandrii*, *M. adhaerens* and *M. aquimarina*.**

**Figure S2.** **Example of Phenoplate Fluorescence measurements with ETR and NPQ calculation.**

**Figure S3: Gating strategy used to collect flow cytometry data.**


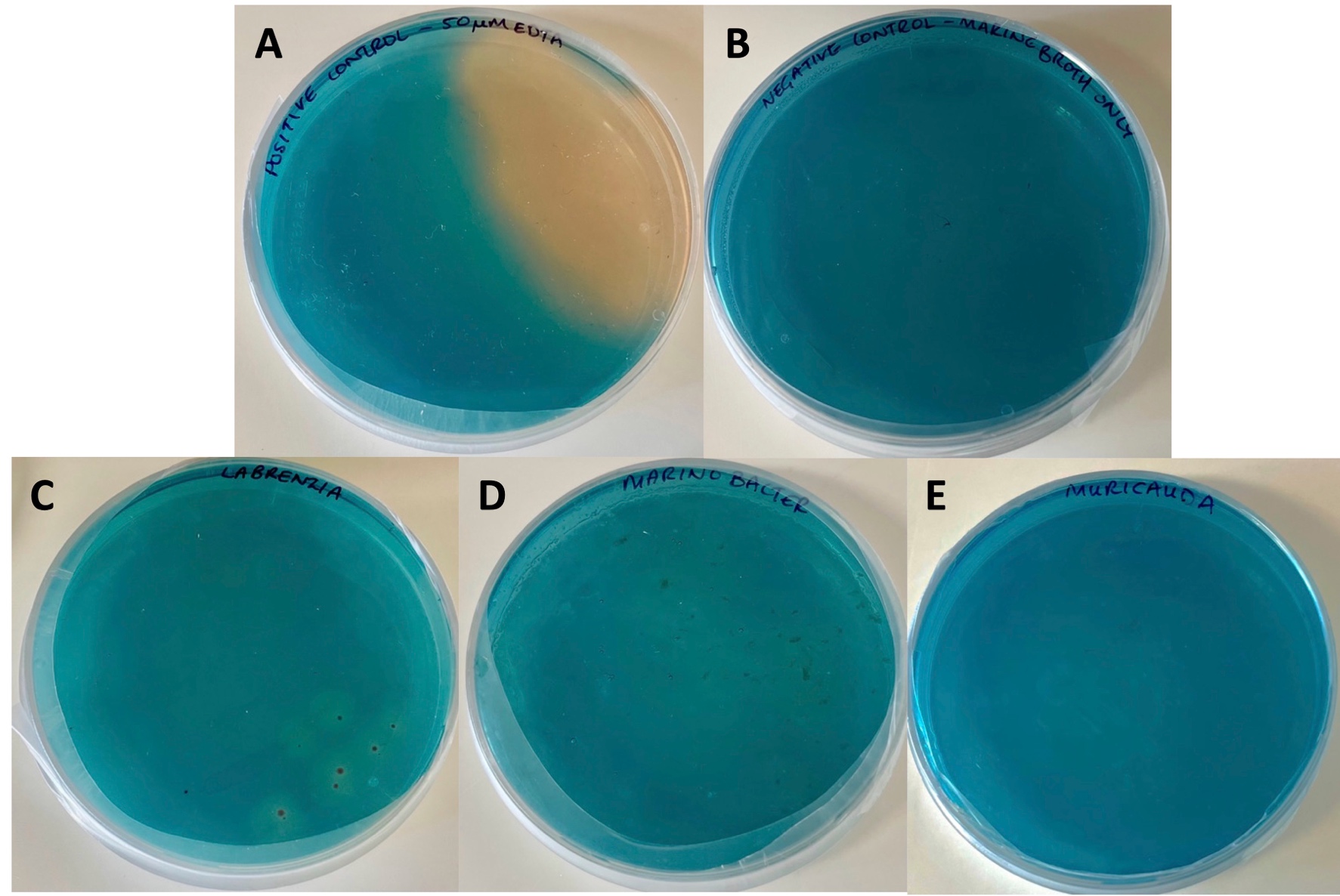


**Figure S1. Iron chelating CAS assay plates for the detection of siderophore production by cultured *L. alexandrii*, *M. adhaerens* and *M. aquimarina*.** Cultures were streaked and incubated for 5 days at 26˚C. Bacteria plates were compared to positive (**A**; 50 µM EDTA generates a yellow discolouration) and negative (**B**; sterile media has no discolouration) control CAS plates. A yellow halo around the bacteria indicated the production of a siderophore in *L. alexandrii* (**C**) and *M. adhaerans* (**D**) plates. No yellow discolouration was observed in the negative control or *M. aquimarina* (**E**) plates.


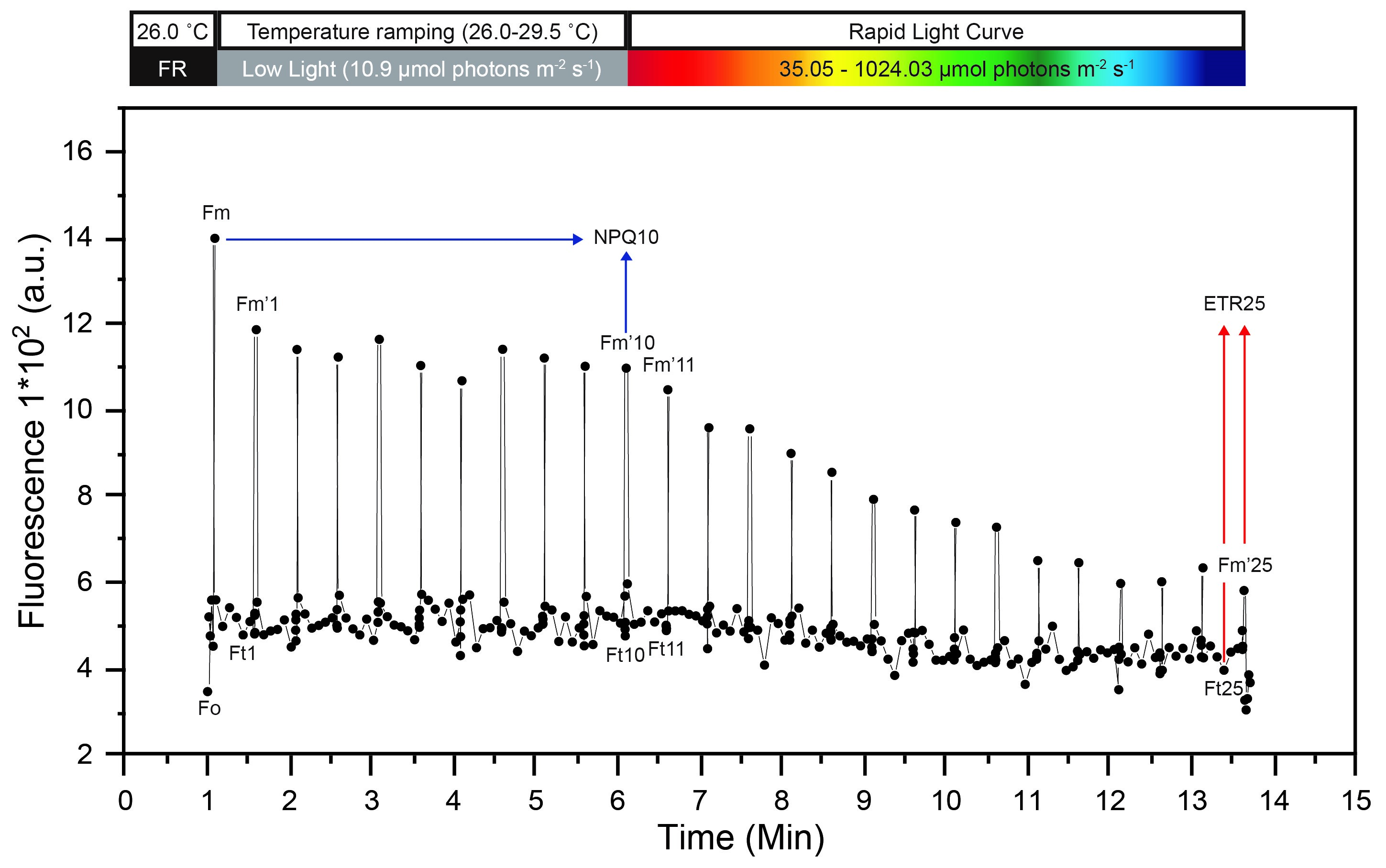


**Figure S2.** **Example of Phenoplate Fluorescence measurements with ETR and NPQ calculation.** Fluorescence tracing (Black line) from phenoplate measurement of one well of culture from the untreated B. minutum culture at 3 days of growth. Each fluorescence point which was used for calculation of photobiological response is numerically numbered 1 though 25, with 1^st^, 10^th^, 11^th^ and 25^th^ peaks marked in the figure. Fm’1 through Fm’10 and Ft1 through Ft10 occur during temperature ramping, Fm’11 to Fm’25 and Ft11 to Ft25 occurred during the rapid light curve. Far red illumination (FR) was applied for 1 minute with control temperature maintained for Fo measurement. Fm measurement was taken after saturating pulse was applied at the 1 minute mark. Temperature ramping proceeded for 5 minutes under low light in which Fm' and Ft was measured every minute. At 6 minutes the rapid light curve proceeded with stepwise increases of light pulses from 35.05-1024.03 µmol photons m-2 s-1, per 30 seconds for Fm'11-Fm'25 and Ft11-Ft25 measurements which were used for calculation of light curve rETR and NPQ. Blue lines indicate the measurements used for calculation of NPQ at each time point using the formula NPQ = ([F_m_-F_m_´]/F_m_´), as specified in figure. Red lines define Fluorescence measurements used to calculate rETR at each time point according to the formula rETR=([Fm'-Ft]/Fm')*PAR*0.5*0.84.

**
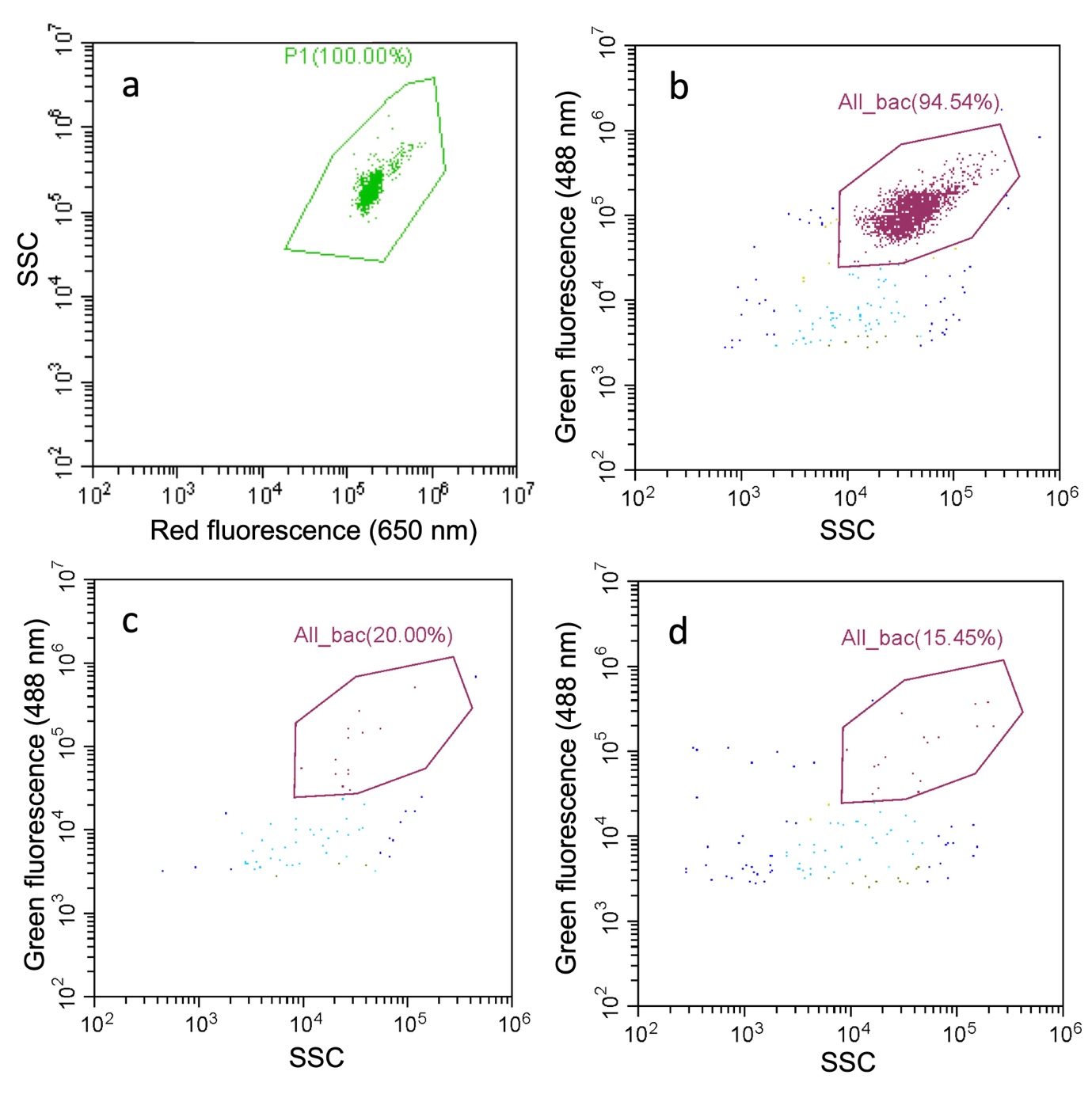
Figure S3: Gating strategy used to collect flow cytometry data. (a)** Bacteria population characterized according to side scatter (SSC) and SYBR Green fluorescence. **(b)** Symbiodiniaceae sp. population characterized according to side scatter (SSC) and red fluorescence (chlorophyll). **(c)** Example bacteria blank (media only) flow cytometry results used for blank corrections. **(d)** Example of EBR treated culture flow cytometry results.
